# Specific histamine binding activity of a new lipocalin from *Hyalomma asiaticum* (Ixodidae) and therapeutic effects on allergic asthma in mice

**DOI:** 10.1186/s13071-016-1790-0

**Published:** 2016-09-17

**Authors:** Yanan Wang, Zhuang Li, Yongzhi Zhou, Jie Cao, Houshuang Zhang, Haiyan Gong, Jinlin Zhou

**Affiliations:** 1 Key Laboratory of Animal Parasitology of Ministry of Agriculture, Shanghai Veterinary Research Institute, Chinese Academy of Agricultural Sciences, Shanghai, 200241 China; 2 Chizhou Vocational and Technique Colleague, Chizhou, 247000 China; 3 Jiangsu Co-innovation Center for Prevention and Control of Important Animal Infectious Diseases and Zoonoses, Yangzhou, 225009 China

**Keywords:** Lipocalin, *Hyalomma asiaticum*, Histamine binding, Allergic asthma in mice

## Abstract

**Background:**

Lipocalin proteins are secreted by tick salivary glands as an important strategy to interfere with the immune response of hosts. A large number of lipocalins are secreted, but the functions of most of these proteins are unclear. Here, we report a new lipocalin protein with particular histamine binding capacity, which was isolated from the salivary glands of the tick *Hyalomma asiaticum*.

**Methods:**

The full length cDNA of the Ha24 gene was obtained by RACE, and Ha24 gene was expressed in *E. coli*; after protein purification and mice immunizations, specific Polyclonal antibodies (PcAb) were created in response to the recombinant protein. Reverse transcription PCR (RT-PCR), Quantitative PCR (Q-PCR), indirect immunofluorescence antibody (IFA) assay and western blot were used to detect the existence of native Ha24 in ticks. To confirm the histamine-binding capacity of rHa24, a histamine-binding assay was completed in vitro (ELISA) and in vivo by inhibition of allergic asthma in mice.

**Results:**

Ha24 is coded by 681 bases, contains 227 amino acids, and has a molecular weight of 23.3 kDa. Abundant expression in the salivary glands of feeding ticks was confirmed by the identification of native Ha24 in ticks. The results of a histamine binding assay both in vitro and in vivo demonstrated that rHa24 binds specifically with histamine in a dose-dependent manner, and can provide relief from allergic asthma in mice.

**Conclusions:**

Ha24 is a new tick lipocalin with specific histamine binding activity that can provide relief from host inflammation response.

## Background

Lipocalin proteins, secreted by tick salivary glands, are involved with the ability of ticks to overcome immunity mechanisms of their hosts [1]. Lipocalin proteins can bind and block the corresponding ligands associated with inflammatory and blood clotting responses such as pheromones, steroids, bilins, retinoids and lipids [2–6]. Lipocalins typically contain 160–180 amino acid residues and low sequence homology but high structural level [7–9]. The conserved structure is the β-barrel, which is usually composed of several antiparallel strands with an end closed by N-terminal peptide segments. More than 300 different lipocalins have been identified from tick salivary glands [10]. Lipocalin genes (more than 40 genes have been found) are evolving more rapidly than other gene families in tick salivary glands and this may be due to immune pressures imposed by their hosts [11]. One representative lipocalin is the Histamine-binding protein (HBP), which was identified from the hard tick *Rhipicephalus appendiculatus*; HBP was the first tick lipocalin identified that possessed an immunomodulatory function that worked by scavenging histamine [12–14]. Many lipocalins, from both hard and soft ticks, bind to different bioactive ligands such as histamine, serotonin, leukotriene B4, leukotriene C4, and thromboxane A_2_, which relate to immuno-modulation and platelet aggregation [3, 9, 15, 16]. Lipocalins also play important roles in tick feeding, and are useful to study the functions of different lipocalins.

Tick histamine-binding proteins and related lipocalins are potential candidates for therapeutic use against various diseases [17]. The rEV-131 lipocalin has been developed for the treatment of asthma, allergic rhinitis and other neutrophil-mediated disorders [18]. The rEV-598 lipocalin from *Dermacentor reticulatus* (Ixodidae) has been used for treatment of carcinoid syndrome and post-chemotherapy emesis [3]. Lipocalin rEV-576 binds to the complement factor C5/C5a and has been tested in many inflammatory models, including rheumatoid arthritis and reperfusion injury; it is currently in preclinical study [19].

*Hyalomma asiaticum* is one of the three hard ticks (Ixodidae) widely distributed in northwest China and Central Asia [20]. These ticks are vectors of *Babesia* spp. and *Theileria* spp. in animals and humans [21]. In this study, we report on a new lipocalin from *H. asiaticum* with specific histamine binding activity and capability to provide relief from host inflammation response.

## Methods

### Total RNA extraction of salivary glands and cloning of the Ha24 gene

Salivary glands from several partially fed adult female *H. asiaticum* ticks were dissected out and placed in sterile phosphate buffer saline. RNA was isolated from these salivary glands using Trizol reagent (Invitrogen, Carlsbad, CA, USA) and cDNA of the salivary gland RNA was synthesized from the purified mRNA using the Reverse Transcription System Kit (Takara, Dalian, China). The full length cDNA of the Ha24 gene was obtained by RACE (Invitrogen), which is a method for cloning the 5' and 3' ends of the gene. From the EST sequences of Ha24 in the differentially expressed gene library of *H. asiaticum* salivary glands, the 3' RACE primers were designed and used to amplify the 3' end. Sequencing of this construct revealed the 3' region of the Ha24 coding sequence. This was then used to design primers for the amplification of the 5' region using the 5' RACE System. After sequencing the 5' and 3' sequences were used to design primers (Forward: 5'-CCA GTG TTT ATC AAG GGT AAA GAT G-3'; Reverse: 5'-TAG AAA TTG TAC CTT CCC ACT TAC-3') for the amplification of the full-length Ha24 coding sequence.

### Expression and purification of Ha24 in the prokaryotic expression system

Two pairs of primers, F-*Eco*RI (5'-GC*G AAT TC*G AGA AAG TAA TGG CTC ACA AAG AGG-3') and R-*Xho*I (5'-CG*C TCG AG*C GAT TTC GGC AAT ATT TTC TTC AAT-3'), designed for the amplification of the ORF of Ha24 gene, contained two restriction sites (underlined). After digestion with *Eco*RI and *Xho*I (New England Biolabs, Ipswich, MA, USA), the coding sequence of Ha24 was ligated into a similarly digested pGEX-4 T-1(Invitrogen) expression vector, which carried the tac promoter, GST tag sequence and ampicillin resistance gene. Then the pGEX-4 T-1-Ha24 was transformed into BL21 (DE3) (TIANGEN, Beijing, China) clones were picked and the plasmids were purified using the AxyPrep™ plasmid Miniprep kit (Axygen, Suzhou, China). For induction of recombinant Ha24 expression, isopropyl thio -β-D -galactoside (IPTG) was added to a final concentration of 1 mM and expression was induced at 20 °C for 12 h. After expression, the recombinant Ha24 (rHa24) was affinity-purified under both native and denaturation conditions using GST agarose and the *AKTA FPLC* protein purification system (GE).

To obtain the polyclonal antibody (PcAb), we cloned the Ha24 gene into pET-30a (Invitrogen) using gene specific primers: F-*Nde*I (5'-AAG GAG ATA TA*C ATA TG*G AGA AAG TAA TGG CTCA CAA AGA GGA G-3') and R-*Xho*I (5'-GGT GGT GGT G*CT CGA G*CG ATT TCG GCA ATA TTT TCT TCA ATT CTT TTT CTG-3'). pET-30a were digested with *Nde*I and *Xho*I (New England Biolabs), and ligated with the Ha24 gene sequence which was amplified by CloneAmp™ HiFi PCR Premix (Clontech, Takara Bio, Palo Alto, CA, USA) using In-Fusion HD Cloning Kits (Clontech, Takara Bio). After the pET-30a-Ha24 was obtained, the next steps were in line with methods above.

### Quantitative PCR (Q-PCR) and reverse transcription PCR (RT-PCR)

The different developmental stages of the tick were attached to the ears of New Zealand White rabbits (SLAC, Shanghai Institutes for Biological Science, CAS). The eggs, larvae, and nymphs of *H. asiaticum* were collected for different periods of expression of Ha24 gene. The adult female or male ticks (fed for 1–7 days) were collected sequentially and the female fed ticks were dissected under the microscope to obtain the salivary glands and midguts. Total RNA was extracted using Trizol reagent (Invitrogen). RNA was converted into first-strand cDNA using a Prime Script RT reagent kit (Perfect Real Time, Takara) according to the manufacturer’s protocol.

All quantitative PCR (Q-PCR) assays were performed using SYBR Premix Ex Taq (Takara) green and Ha24 gene specific primers (F: 5'-GAC TGG CGA TGA CAA AGT TCT CCC-3' and R: 5'-CCG AGC ATT ATC GTT TTC CAC CAG-3'), ELF1A [22] reference gene specific primers (F: 5'-CGT CTA CAA GAT TGG TGG CATT-3' and R: 5'-CTC AGT GGT CAG GTT GGC AG-3') with a Step One Plus Real-Time PCR System (Applied Biosystems, New York, New York, USA), with cycling parameters of 95 °C for 30 s, followed by 40 cycles at 95 °C for 5 s, and 60 °C for 30 s. All samples were analyzed three times.

The data were normalized to ELF1A Relative gene expression data were analyzed using the 2 ^-△Ct^ method; △Ct values were calculated by subtracting the average ELF1A Ct values from those for the average target gene.

### Antibody production, western blot and IFA assay

rHa24-His was produced in *E. coli* (100 μg) and Freund’s adjuvant (complete and incomplete Freund’s adjuvant, Invitrogen) were emulsified in equal volume proportions. The mixture was injected into 6–8 week-old BALC/c mice (SLAC, Shanghai Institutes for Biological Science, CAS) 3 times at 2-week intervals with the same dose of antigen. Serum was collected 3 days after the third injection.

Western blot was used to analyze the native Ha24 expression in feeding adult female tick salivary glands. The salivary glands were collected using a microanatomic method. Sodium dodecyl sulfate polyacrylamide gel electrophoresis (SDS-PAGE) was performed using 12 % Express Plus™ PAGE Gels (GenScript, Nanjing, China) and the separated proteins were transferred to a PVDF membrane (Millipore) using 13 V for 33 min in a blotting buffer (0.1 M Tris, 192 mM glycine) with 20 % methanol. Membranes were blocked with PBS (PH 7.4, with 0.14 M NaCl and 0.0027 M KCl 0.01 M phosphate buffer containing 5 % skim milk), initially probed with rHa24 PcAb, followed by HRP-Goat anti Mouse IgG, and then detected with DAB Staining Kit (TIANGEN).

The location of Ha24 in the tick salivary glands was analyzed using the IFA assay. The salivary glands were collected on feeding day 4 and then fixed in 4 % paraformaldehyde. After paraffin-sectioning, the sections were permeabilized with 0.2 % Triton X-100 and incubated for 1 h with 1:100 mouse anti-Ha24 PcAb in buffer PBS/2 % bovine serum albumin. After antibody removal and three washes with PBS/0.5 % Tween-20, FITC-Goat anti Mouse IgG (1:1000, Life Technologies, Carlsbad, CA, USA) antibodies were detected by incubating the salivary glands for 30 min. For nuclear staining, sections were incubated with 1 μg/ml 4', 6'-diamidino-2-phenylindole (DAPI, Invitrogen) in dd H_2_O for 20 min. After washing, the sections were mounted using fluorescent mounting medium under glass coverslips, then viewed and photographed on the Axio Observer Z1 Epifluorescence Microscope (Zeiss).

### Histamine binding assays using ELISA

rHa24-GST and GST were diluted to 10 μg/ml, 5 μg/ml, 2.5 μg/ml and 1.25 μg/ml concentrations with 0.05 M carbonate buffer (pH = 9.6). A 100 μl sample of these dilutions was added to each well of a 96-well Maxisorp plate, and each concentration corresponding to three well replicates. The plate was incubated at 4 °C for 8 h. Then, the wells were rinsed three times using 100 μl/well of wash buffer. Moisture was removed by blotting with a paper towel. The wells were then blocked with 3 % skim milk in 0.05 % Tween-20 in phosphate buffer at 37 °C for 1 h. The solution was discarded and the wells were rinsed three times using the methods described above. Histamine-HRP (Neogen Veratox®, Lansing, MI, USA) were dispensed into wells (100 μl/well) and incubated at 37 °C for 2 h. After washing the wells three times, 100 μl of well-mixed substrate was added to each well. The reaction proceeded for 10 min, and then 100 μl of red stop solution was added to the wells. The OD values were measured at 650 nm.

### Murine allergic asthma model and treatment

Six to eight week old female BALC/c mice, obtained from SLAC, were immunized twice, at weekly intervals, with 0.4 ml saline containing 100 μg OVA (Ovalbumin, Sigma-Aldrich, St Louis, MO, USA) mixed with 0.2 ml Freund’s Complete Adjuvant (F5881-10 ML; Sigma-Aldrich). For subsequent injections, the Ag dose was reduced to 50 μg of OVA per injection. One week after the second immunization at day 14, an intranasal challenge was performed under light ether by applying 50 μl of OVA in saline solution (10 μg) or saline alone as a control [18]. rHa24-GST, GST (negative control) and Mepyramine (340 μg, m.w. 402; Sigma-Aldrich; positive control), was administered intratracheally with 50 μl saline buffer under ketamine anesthesia, 1 h before the OVA challenge. BAL (Bronchoalveolar lavage) was performed 3 days after the intranasal challenge by cannulating the trachea under ketamine anesthesia and washing two times with 1 ml ice-cold PBS. The BAL was centrifuged, and the supernatant was frozen for histamine. The cell pellet was re-suspended in PBS and counted by a hemocytometer.

After BAL, the mice were sacrificed (3 days after OVA challenged). The whole lung was removed and fixed in 4 % paraformaldehyde for Hematoxylin & Eosin and Periodic acid Schiff reagent staining.

### Data analysis

Graphpad PRISM 5 software (GraphPad Software Inc., La Jolla, California) was used for data analysis. Mean ± standard error (SEM) values for each group were calculated, and two-tailed *t*-tests were used to detect differences between groups.

## Results

### Ha24 cloned from the cDNA of *Hyalomma asiaticum* salivary glands

**Fig. 1:**
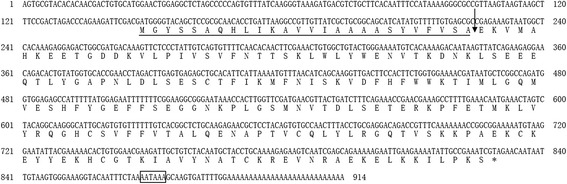
Nucleotide sequence and deduced amino acid sequence of Ha24. Start codon ATG, stop codon TAG and polyadenylation signal AATAAA are boxed; the sequence of the transmembrane is underlined; the arrow indicates the putative cleavage point for the release of the signal peptide

**Fig. 2:**
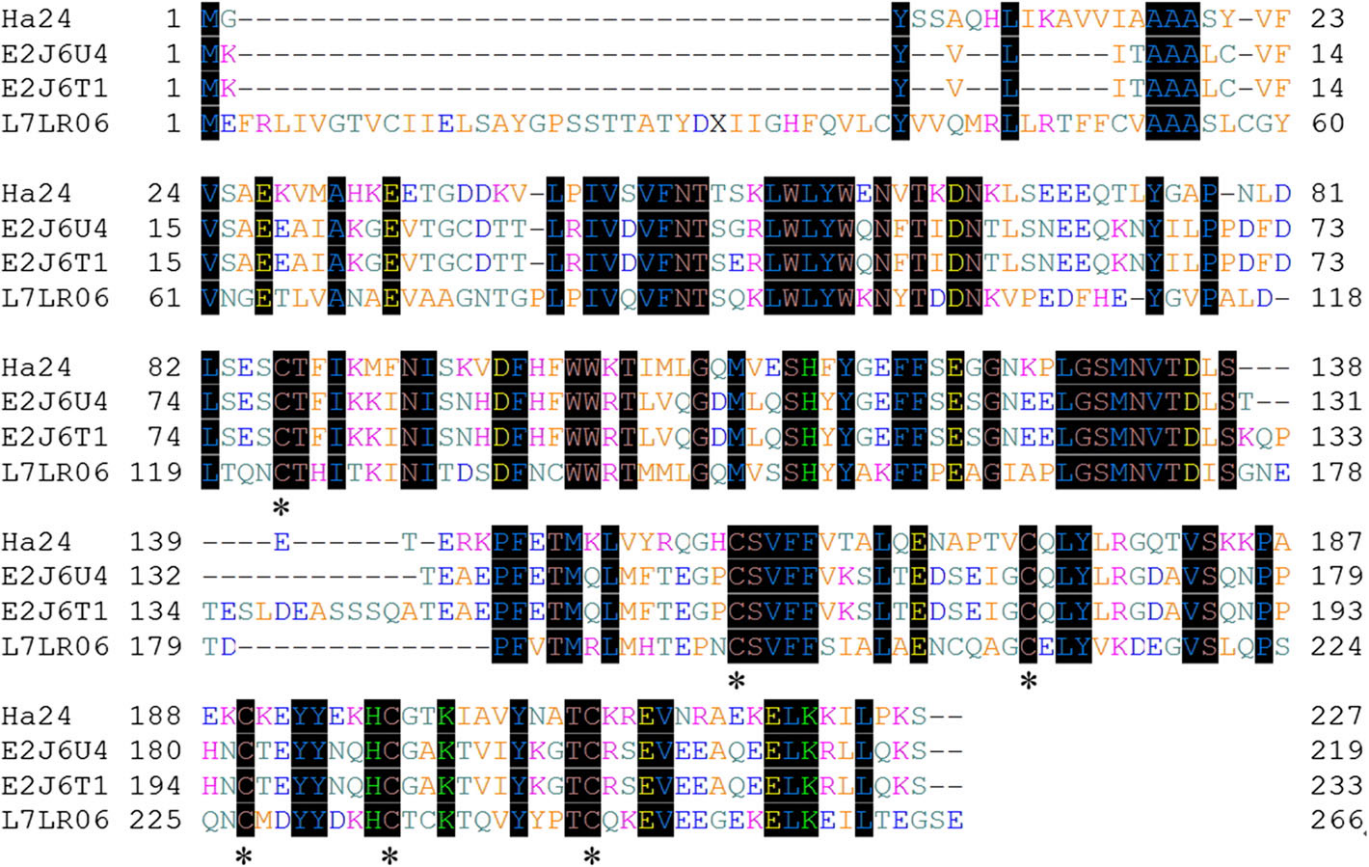
Amino acid sequence homology of *Hyalomma asiaticum* Ha24 to lipocalins. *Key*: Ha24: the new lipocalin amino acid sequence of *H. asiaticum*; E2J6T1: amino acid sequence of *Hyalomma marginatum rufipes* lipocalin group II group II b; E2J6U4: amino acid sequence of *H. marginatum rufipes* lipocalin group IV subgroup metastriate; L7LR06: amino acid sequence of *Rhipicephalus pulchellus* Putative group *v* salivary lipocalin

Ha24 (GenBank accession no. KX618193) consisted of 681 bases that encoded a 227 amino acid peptide (Fig. 1). The sequence was analyzed by SignalP 4.1 (http://www.cbs.dtu.dk/services/SignalP/) and ExPASy (http://web.expasy.org/compute pi/; http://prosite.expasy.org/), which suggested that it is a secretory protein comprised of a 26 residue, cleavable signal peptide followed by a 201 residue mature peptide of predicted 23.3 kDa molecular weight, a theoretical pI of about 7.79 and contain 9 phosphorylation sites, 5 N-glycosylation sites and 2 N-myristoylation sites according to the prosite analysis. The primary amino acid sequence indicated that Ha24 is a member of the lipocalin family and shows similarities to other lipocalins (Fig. 2). This determination was made because of the conserved number and position of the cysteine residues [23].

**Fig. 3:**
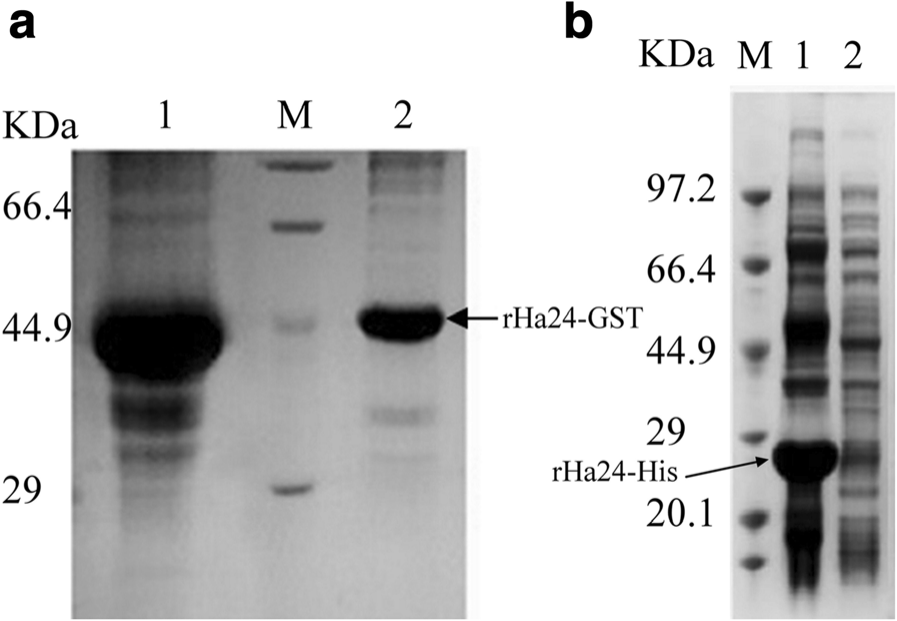
Expression of rHa24. **a** Expression of rHa24-GST. Lane 1, IPTG induced supernatant of cell pellet (pGEX-4 T-1-rHa24-GST) after sonication; Lane M, the protein marker; Lane 2, rHa24-GST after purification. **b** Expression of rHa24-His. Lane M, the protein marker; Lane 1, IPTG-induced supernatant of cell pellet (pET-32A-rHa24-His) after sonication; Lane 2, rHa24-His after purification

The coding sequence of Ha24 was subcloned into prokaryotic expression vectors (pGEX-4 T-1 and pET-30a) and then recombinant rHa24-GST and rHa24-His were obtained. rHa24-GST (~49.9 kDa, Fig. 3a), was soluble, isolated from the supernatant of the *E. coli* and used for the protein function experiments. rHa24-His (~23.8 kDa, Fig. 3b) was insoluble and used for preparation of PcAb from mice. The polyclonal antibodies were characterized by western blot for the specific recognition of their recombinant protein antigens (data not shown).

### Identification of Ha24 in *Hyalomma asiaticum’s* salivary

**Fig. 4:**
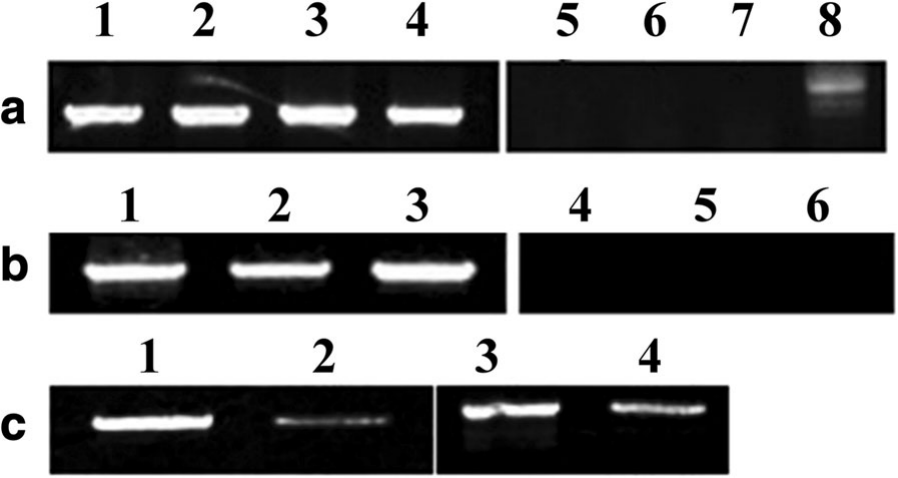
Specific expression of Ha24 in *Hyalomma asiaticum*. **a** Analysis of specific expression of Ha24 gene according to the sex. Lanes 1–4, 5–8: the cDNA of unfed male adult ticks, unfed female adult ticks, fed male adult ticks and partially fed female adult ticks (Lanes 1–4: actin primers; Lanes 5–8: Ha24 gene specific primers). **b** Expression of Ha24 in different development periods. Lanes 1–3, 4–6: the cDNA of eggs, larvae, and nymphs (Lanes 1–3: actin primers; Lanes 4–6: Ha24 gene specific primers). **c** Expression of Ha24 in the salivary glands and midguts of *H. asiaticum*. Lanes 1–2, salivary glands and midguts with actin primers, respectively; Lanes 3–4, salivary glands and midguts with Ha24 gene specific primers, respectively

**Fig. 5:**
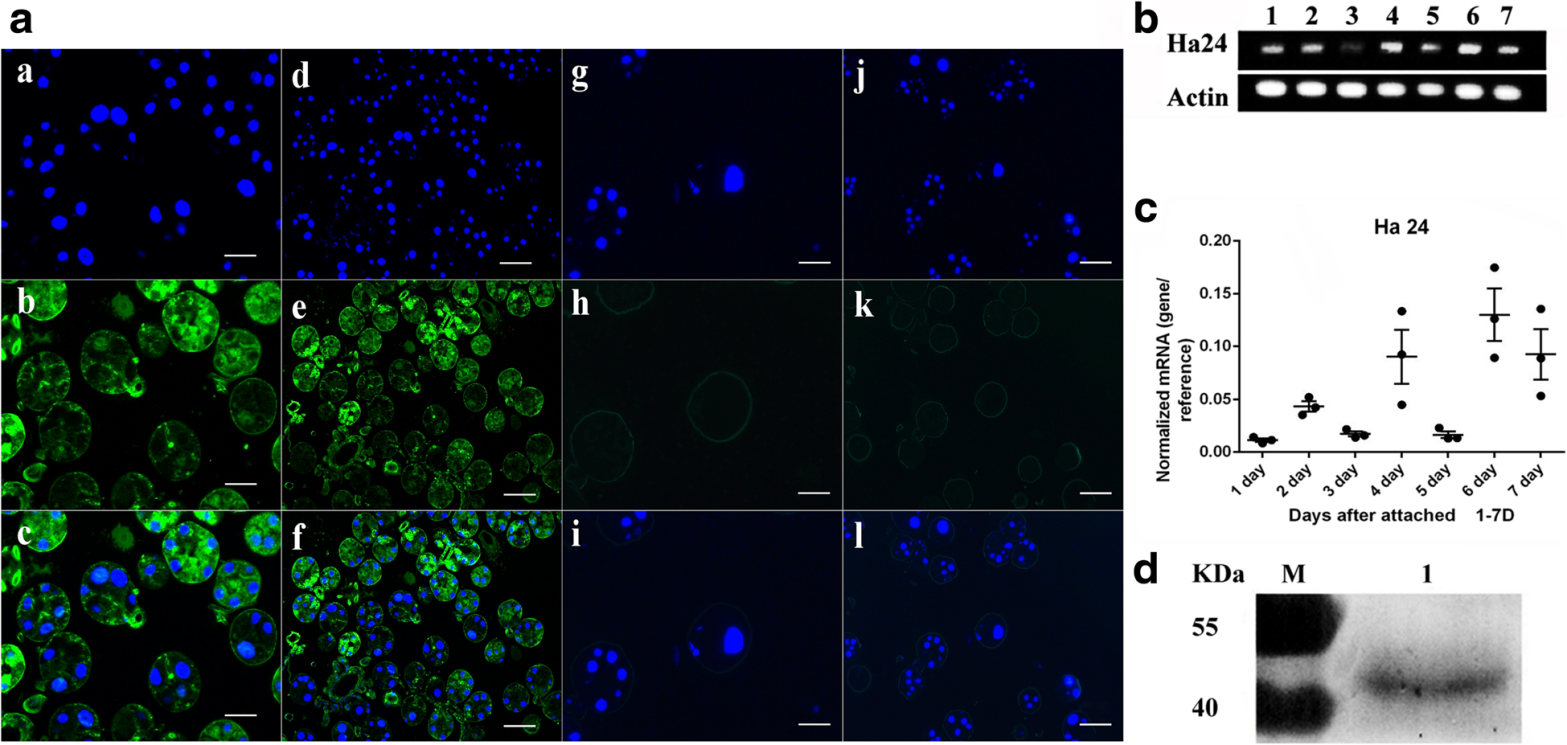
Identification of native Ha24. **a** Immunofluorescence analysis (IFA) of Ha24 in feeding tick salivary glands. Tick salivary glands were stained with mouse anti-Ha24 PcAb (**a**-**f**) and wild mouse PcAb (control, **g**-**l**), and DNA were stained with DAPI. **a**, **d**, **g**, **j** were observed in blue fluorescent (DAPI); **b**, **e**, **h**, **k** were observed in green fluorescent (FITC); **a** and **d**, **b** and **e**; **g** and **h**; and **j** and **k** were merged (shown in **c**, **f**, **i** and **l**, respectively). *Scale-bars*: **a**-**c**, **g**-**i**, 100 μm; **d**-**f**, **j**-**l**, 50 μm. **b** RT-PCR analysis of Ha24 in tick salivary glands (1–7 days after attached). 1 % agarose gel showing the Q-PCR products from feeding female tick salivary glands. Lanes 1–7: cDNA of tick salivary glands at different feeding periods with Ha24 specific primers; Lanes 1–7 below: cDNA of tick salivary glands at different feeding periods with tick actin primers. **c** Q-PCR analysis of Ha24 in tick salivary (1–7 days after attachment). The relative expression of Ha24 mRNA *vs* that of actin mRNA was investigated by quantitative reverse transcription polymerase chain reaction in different tick feeding stages. **d** Identification of native Ha24 by western blot. Lane M, pre-stained protein ladder; Lane 1, anti-Ha24 PcAb from feeding female tick salivary glands

The cDNA of eggs, larvae and nymphs were subjected to RT-PCR for the different specific periods of expression of Ha24 gene (Fig. 4b). The cDNA of unfed and fed adult (female and male) ticks was used for the sex-specific expression of the Ha24 gene (Fig. 4a). After microdissection, different organs of adult female ticks were obtained, such as salivary and midgut, whose cDNA was used for the expression detection of the Ha24 gene (Fig. 4c). The cDNA of (1–7 days) feeding tick salivary glands were subjected to RT-PCR and Q-PCR analysis (Fig. 5b, c). The results demonstrated that Ha24 was expressed in whole feeding stages (including the early feeding period, the fast feeding period, and the end of the feeding period) of ticks and the transcription level of Ha24 varied.

Anti-rHa24 PcAb was prepared from mice and used to confirm the presence of native Ha24 in the salivary glands from feeding adult female ticks. Ha24 was detected by immunofluorescence in the 4 day-fed tick salivary glands; in the serum of healthy mice it was detected by the same methods used for the control (Fig. 5a). The results showed that Ha24 was mostly present in the feeding tick salivary glands, and the type III acinus and duct were strongly positive for Ha24.

Western blot was used to determine the molecular weight of rHa24 and demonstrated that native Ha24, with a molecular weight about 48 kDa, was present in the tick salivary glands (Fig. 5d). The molecular weight of native Ha24 was greater than expected based on its amino acid sequence, which may be due to its various phosphorylation sites and N-glycosylation sites.

### Ha24 specifically binds histamine

**Fig. 6:**
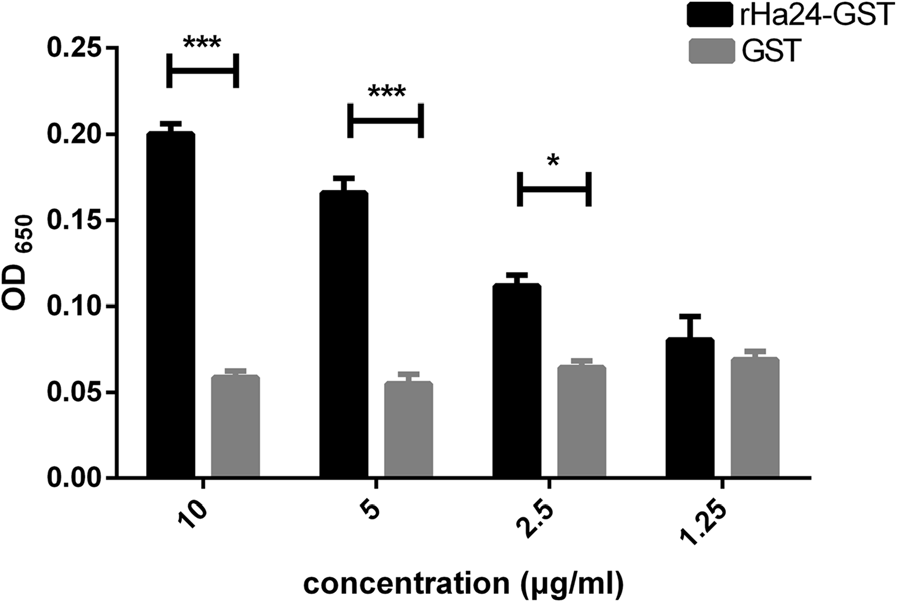
The special binding assay between rHa24 and histamine. The binding capacity between Ha24 (*black*) and histamine was changed with different concentrations (10, 5, 2.5, 1.25 μg/ml) of rHa24, and GST (*gray*) in the same concentrations as control. Mean values and standard errors (SEM) are given from two independent experiments. Each concentration was tested 3 times and differences were significant at *P < 0.05; **P < 0.01; ***P < 0.001

Different concentrations of rHa24-GST and GST were incubated with histamine-HRP, and the OD value was measured at 650 nm (Fig. 6). The experiment methods followed the instructions of the Veratox® (Neogen, Lansing, MI, USA) for histamine and above. The results showed that rHa24 has the ability to bind with histamine and the histamine binding force was reduced following the concentration reduction of rHa24 (GST, as control, 10 μg/ml: *t*_(3)_ = 19.96, *P* < 0.0001; 5 μg/ml: *t*_(3)_ = 11.02, *P* = 0.0004; 2.5 μg/ml: *t*_(3)_ = 6.52, *P* = 0.0029; 1.25 μg/ml: *t*_(3)_ = 0.79, *P* = 0.4722).

### Ha24 inhibits allergic asthma of mice

**Fig. 7:**
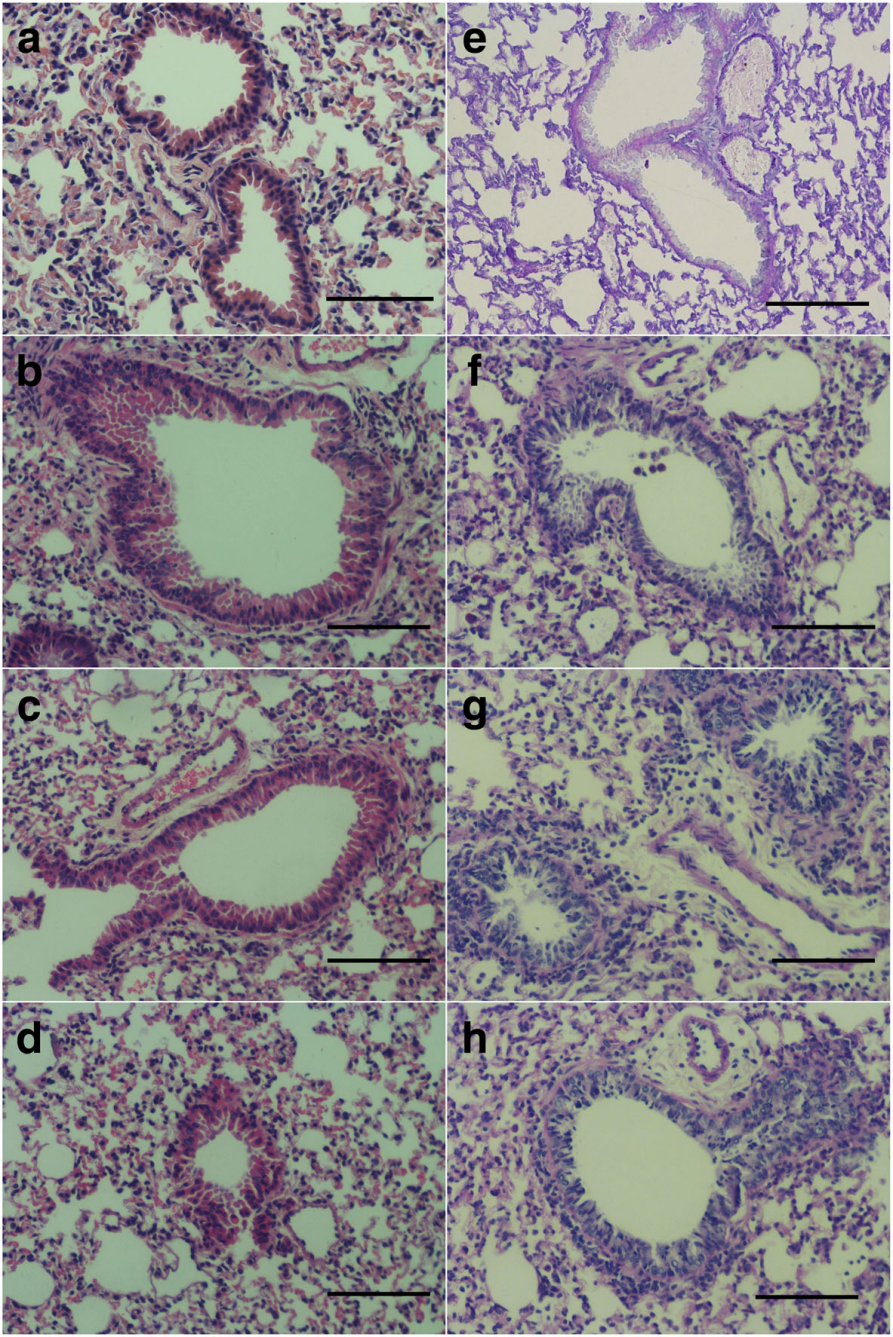
rHa24 inhibits inflammation and protein secretion. **a**-**d** Hematoxylin & eosin staining results. **a** Normal lungs. **b** Inflammatory changes of OVA-GST challenged mice. **c**, **d** rHa24-GST and Mepyramine mice with improvement of pathological changes in the lung tissue and reduction of inflammatory cell infiltration. **e**-**h** Periodic acid Schiff reagent staining. **e** Normal lungs. **f** Hypersecretion changes of OVA-GST challenged mice. **g**, **h** rHa24-GST and Mepyramine with inhibition of secretion in mice. *Scale-bars*: 100 μm

After methacholine (Sigma-Aldrich) aerosolized for 1 min, no obvious symptoms in rHa24 and Mepyramine groups, but different degrees of clinical symptoms, characterized by irritability, scratched ears, breathlessness, incontinence and other symptoms emerged in OVA-GST group. OVA-challenge caused severe lung tissue damage and inflammatory cell infiltration. By contrast, Mepyramine and rHa24 improved the pathological changes of lung tissue, as well as resulted in reduced inflammatory infiltration and tissue destruction (Fig. 7a-c). OVA-challenge also caused significant hyperplasia and mucus hypersecretion, both symptoms arising in the bronchial smooth muscle and goblet cells. But Mepyramine and rHa24 improved the OVA-induced symptoms (Fig. 7d-f).

**Fig. 8:**
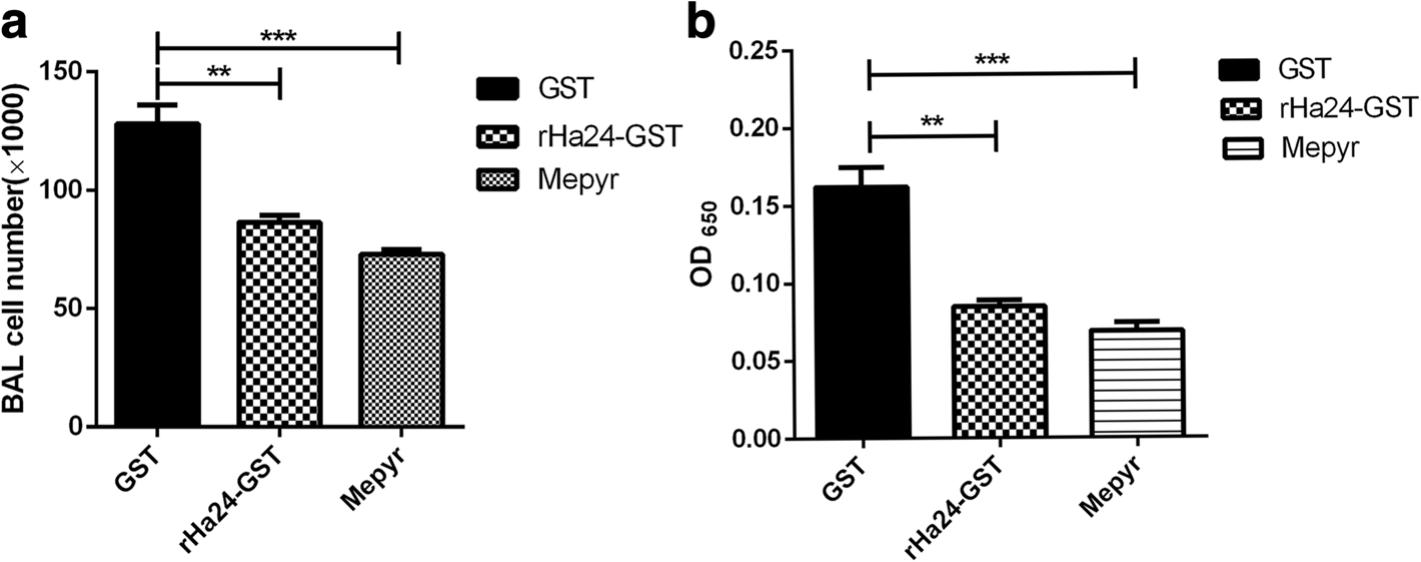
Effects of rHa24 on the number of total cells and secretion of histamine in BAL. **a** Effects of rHa24 on the number of total cells in BAL from mice with OVA-GST induced allergic asthma. rHa24 and Mepyramine inhibited the number of OVA-GST induced inflammatory cells. **b** Effects of rHa24 on the secretion of histamine in BAL from mice with OVA-induced allergic asthma. rHa24 and Mepyramine inhibit the secretion of OVA-GST induced histamine. The results are from two independent experiments; mean values and standard errors (SEM) are given (*n* = 4 mice per group) and differences were significant at *P < 0.05; **P < 0.01; ***P < 0.001

OVA-challenge caused a substantial recruitment of inflammatory cells and secretion of histamine into the BAL fluid. The results demonstrating the number of leukocytes in each sample are shown in Fig. 8a. The leukocytes’ (mainly including eosinophils and neutrophils) number of OVA-GST groups increased significantly compared to the other two groups; Mepyramine groups and rHa24 groups significantly inhibited the production of OVA-induced leukocytes. OVA-GST was challenged compared with rHa24 group (*t*_(4)_ = 4.727, *P* = 0.0032) indicated a profound difference; OVA-challenged compared with the Mepyramine group (*t*_(4)_ = 6.466, *P* = 0.0006) showed a highly important difference.

We also investigated the secretion of histamine in different groups. There was a significant increase in the secretion of the histamine in OVA-challenged groups compared to the other two groups, and the Mepyramine group and rHa24 group significantly inhibited OVA-induced histamine secretion (Fig. 8b). OVA-GST challenged groups were compared with rHa24 groups and the difference was significant (*t*_(4)_ = 5.771, *P* = 0.0012); OVA-GST challenged group were compared with Mepyramine group and the difference was highly remarkable (*t*_(4)_ = 6.717, *P* = 0.0005).

## Discussion

The number of tick protein sequences in the databases is relatively limited. This hinders identification of tick proteins using only proteomics. Species- or organ-specific EST libraries make it possible to discover more genes with differential expression [24, 25]. We cloned and expressed a new gene from the salivary gland of feeding *H. asiaticum.* Using the conserved number and position of cysteine residues, we identified this gene as a lipocalin and named it “Ha24”. A histamine-binding assay using ELISA confirmed that rHa24 can bind histamine. The allergic asthma mice model was used for its ability to bind histamine in vivo. Results confirmed our prediction of binding but the binding mechanism was not studied.

Ha24 was specific to female ticks and not found in other developmental stages (Fig. 4a-c). Ha24 expression was not limited to the salivary glands, and also occurred in other organs of adult female feeding ticks, such as the midgut. The highest expression was in the salivary glands. Q-PCR and RT-PCR detection results showed that Ha24 expression occurs throughout the feeding period but at variable levels. Expression may be related to the intermittent immune response of hosts, and this provides evidence that Ha24 is an immune-related molecule. The expression of Ha24 are similar to Ra-HBP3 (which is the male-specific histamine binding protein) as well as other HBPs usually found in the early feeding period [12].

Histamine binding experiments confirmed the dose-dependent histamine binding capacity of rHa24, while the control group GST did not bind at any concentration. In these experiments, the use of ELISA, as an assay for detection of binding between rHa24 and histamine, is new. However, this experiment did not reveal the essential histamine binding characteristics of rHa24, such as binding constants, enthalpy change (ΔH), and dissociation constants [26]. Additional experiments will be needed to reveal the Ha24 binding mechanism.

The histamine binding protein rEV131 significantly inhibits allergic asthma in mice [18]. We selected allergic asthma in mice as a method for studying histamine binding of rHa24 in vivo, and used Mepyramine (histamine H1 receptor antagonist) as the positive control. According to Couillin et al. [18] Mepyramine efficiently inhibits allergic asthma by scavenging histamine. Our results showed that Ha24 can also inhibit allergic asthma in mice, partly confirming histamine binding of Ha24. But we still need follow-up experiments to make sure the inhibition mechanism of Ha24 in allergic asthma, which may cause by the histamine-binding or others. Four unique receptor (HR1, HR2, HR3 and HR4) subtypes can mediate the production of histamine. HR1 and HR2 always express on lymphoid and nonlymphoid cells [27] and are associated with the inflammatory response. HR1 antagonists have been used to treat allergy but always in combinations because of their individual limitations in mediating histamine [28]. Ha24 and other HBPs can block histamine and neutralize its effects. In this way, it may be more efficient than traditional HR antagonists with fewer side effects.

## Conclusion

We confirmed the histamine binding ability of Ha24 both in vitro and in vivo, but we lack information on its mode of action, such as its composition binding sites and the existence of other possible ligands which can also be blocked by Ha24. We will continue the study of the competitive histamine binding mechanism between Ha24 and several other histamine receptors (HR1, HR2, HR3 and HR4), and will perform a histamine-binding comparison with other histamine-binding proteins.

## Abbreviations

BAL, Bronchoalveolar lavage; DAPI, 4', 6'-diamidino-2-phenylindole; HBP, Histamine-binding protein; IFA, Immunofluorescence antibody; IPTG, Isopropyl β-D-1-Thiogalactopyranoside; OVA, Ovalbumin; PBS, Phosphate-buffered saline; PcAb, Polyclonal antibodies; Q-PCR, Quantitative PCR; SDS-PAGE, Sodium dodecyl sulfate polyacrylamide gel electrophoresis; SLAC, Shanghai Institutes for Biological Science, CAS
